# Response to treatment of myasthenia gravis according to clinical subtype

**DOI:** 10.1186/s12883-016-0756-3

**Published:** 2016-11-17

**Authors:** Tetsuya Akaishi, Yasushi Suzuki, Tomihiro Imai, Emiko Tsuda, Naoya Minami, Yuriko Nagane, Akiyuki Uzawa, Naoki Kawaguchi, Masayuki Masuda, Shingo Konno, Hidekazu Suzuki, Hiroyuki Murai, Masashi Aoki, Kimiaki Utsugisawa

**Affiliations:** 1 Department of Neurology, Tohoku University Graduate School of Medicine, Sendai, Japan; 2 Department of Neurology, Sendai Medical Center, Sendai, Japan; 3 Department of Neurology, Sapporo Medical University, Sapporo, Japan; 4 Department of Neurology, Hokkaido Medical Center, Sapporo, Japan; 5 Department of Neurology, Hanamaki General Hospital, 4-28 Kajoh-chou, Hanamaki, 025-0075 Japan; 6 Department of Neurology, Chiba University Graduate School of Medicine, Chiba, Japan; 7 Department of Neurology, Tokyo Medical University, Tokyo, Japan; 8 Department of Neurology, Toho University School of Medicine, Tokyo, Japan; 9 Department of Neurology, Kinki University School of Medicine, Osaka, Japan; 10 Department of Neurological Therapeutics, Graduate School of Medical Sciences, Kyushu University, Fukuoka, Japan

**Keywords:** Classification, Cluster analysis, Myasthenia, Onset age, Treatment

## Abstract

**Background:**

We have previously reported using two-step cluster analysis to classify myasthenia gravis (MG) patients into the following five subtypes: ocular MG; thymoma-associated MG; MG with thymic hyperplasia; anti-acetylcholine receptor antibody (AChR-Ab)-negative MG; and AChR-Ab-positive MG without thymic abnormalities. The objectives of the present study were to examine the reproducibility of this five-subtype classification using a new data set of MG patients and to identify additional characteristics of these subtypes, particularly in regard to response to treatment.

**Methods:**

A total of 923 consecutive MG patients underwent two-step cluster analysis for the classification of subtypes. The variables used for classification were sex, age of onset, disease duration, presence of thymoma or thymic hyperplasia, positivity for AChR-Ab or anti–muscle-specific tyrosine kinase antibody, positivity for other concurrent autoantibodies, and disease condition at worst and current. The period from the start of treatment until the achievement of minimal manifestation status (early-stage response) was determined and then compared between subtypes using Kaplan-Meier analysis and the log-rank test. In addition, between subtypes, the rate of the number of patients who maintained minimal manifestations during the study period/that of patients who only achieved the status once (stability of improved status) was compared.

**Results:**

As a result of two-step cluster analysis, 923 MG patients were classified into five subtypes as follows: ocular MG (AChR-Ab-positivity, 77%; histogram of onset age, skewed to older age); thymoma-associated MG (100%; normal distribution); MG with thymic hyperplasia (89%; skewed to younger age); AChR-Ab-negative MG (0%; normal distribution); and AChR-Ab-positive MG without thymic abnormalities (100%, skewed to older age). Furthermore, patients classified as ocular MG showed the best early-stage response to treatment and stability of improved status, followed by those classified as thymoma-associated MG and AChR-Ab-positive MG without thymic abnormalities; by contrast, those classified as AChR-Ab-negative MG showed the worst early-stage response to treatment and stability of improved status.

**Conclusions:**

Differences were seen between the five subtypes in demographic characteristics, clinical severity, and therapeutic response. Our five-subtype classification approach would be beneficial not only to elucidate disease subtypes, but also to plan treatment strategies for individual MG patients.

## Background

Myasthenia gravis (MG) is a neurological disorder that manifests as fatigable and fluctuating weakness of voluntary muscles, which are mediated by autoantibodies against neuromuscular junction proteins in skeletal muscle that impair neuromuscular transmission [1]. MG typically involves the ocular, bulbar, and extremity muscles, and, in severe cases, respiratory muscles. The clinical course and outcome in MG are affected by several different autoantibodies, thymic abnormalities, onset age and disease severity, as well as response to treatment [2–4]. MG is distinguished according to the production of pathogenic autoantibodies such as anti-acetylcholine receptor antibody (AChR-Ab) and anti–muscle-specific tyrosine kinase antibody (MuSK-Ab) [1, 5, 6]. Clinically, MG is often classified into the following three subtypes based on thymic abnormalities and onset age: thymoma-associated MG; early-onset MG (onset age <50 years); and late-onset MG (onset age ≥50 years) [7]. Furthermore, discrimination is observed in the clinical setting—for example, between ocular and generalized MG—based on the distribution of symptoms.

Previously, we reported classifying MG into the following five subtypes using two-step cluster analysis of a detailed cross-sectional data set of 640 consecutive patients (Japan MG Registry Study 2012): ocular MG; generalized thymoma-associated MG; generalized MG with thymic hyperplasia; generalized AChR-Ab-negative MG; and generalized AChR-Ab-positive MG without thymic abnormalities [8]. However, this five-subtype classification approach requires further confirmation, and its clinical relevance remains to be established.

Therefore, in 2015, we conducted a larger cross-sectional survey to obtain clinical parameters from 1,088 consecutive MG patients. In the present study, using this new data set, we attempted to confirm the reproducibility of our five-subtype classification approach and to specify additional characteristics of these five subtypes with a particular focus on response to treatment in the clinical setting.

## Methods

### Patients and clinical factors

This survey was conducted by the Japan MG Registry Study Group, which comprises 13 neurological centers (Table 1). We evaluated patients with established MG between April and July 2015. To avoid potential bias, we enrolled consecutive patients over a short duration (4 months). All 1088 of these MG patients visited our hospitals, provided written informed consent, and underwent analysis. Among these 1088 patients, 331 (30.4%) were included in our previous survey in 2012 [8].

**Table 1:** Institutions participating in the Japan MG Registry Study 2015

Department of Neurology, Sapporo Medical University Hospital, Sapporo
Department of Neurology, Hokkaido Medical Center, Sapporo
Department of Neurology, Hanamaki General Hospital, Hanamaki
Department of Neurology, Sendai Medical Center, Sendai
Department of Neurology, Tohoku University Graduate School of Medicine, Sendai
Chiba Neurology Clinic, Chiba
Department of Neurology, Chiba University School of Medicine, Chiba
Department of Neurology, Tokyo Medical University, Tokyo
Department of Neurology, Toho University Medical Center Oh-hashi Hospital, Tokyo
Department of Neurology, Tokyo Women's Medical University, Tokyo
Department of Neurology, Kinki University School of Medicine, Osaka
Department of Neurology, Graduate School of Medical Sciences, Kyushu University, Fukuoka
Department of Neurology and Strokology, Nagasaki University Hospital, Nagasaki

*Abbreviation*: *MG* myasthenia gravis

The following clinical parameters were obtained for all patients: sex; age; age at disease onset; duration of disease; duration of immunotherapy; history of bulbar symptoms; presence of thymoma or thymic hyperplasia in thymectomized patients; presence of serum AChR-Ab or MuSK-Ab; and presence of other non-MG-specific autoantibodies, such as anti-nuclear antibody, SS-A/SS-B antibody, TSH-receptor antibody, anti-thyroglobulin/thyroperoxidase antibody, and rheumatoid factor. In addition, the current and past disease status and details of treatment were surveyed for all patients. Clinical severity at the worst condition was determined according to the classification of the MG Foundation of America (MGFA) [9], and, in some patients, the MGFA quantitative MG score (QMG) [9, 10] from medical records. Clinical severity at the current condition was determined according to QMG and MG Composite (MGC) scores [11]. Furthermore, all patients completed the Japanese version of the 15-item Myasthenia Gravis Quality-of-Life Scale (MG-QOL-15), [12, 13] upon study entry.

Prednisone and prednisolone are the global standard oral corticosteroids used to treat MG, and prednisolone is generally used in Japan. Therefore, the current use, peak dose [mg/day], and duration of prednisolone ≥20 mg/day were recorded for all patients, as was the use of calcineurin inhibitors, azathioprine, plasmapheresis, and intravenous immunoglobulin.

Finally, the courses of current and past MGFA post-intervention statuses, particularly the time required to achieve first minimal manifestations (MM) or better status lasting more than one month (MM-or-better ≥1 M) [9], were determined as benchmarks for evaluating response to treatment in each patient. These clinical data were fully collected from 923 (84.8%) of the 1088 patients.

### Two-step cluster analysis

To examine the reproducibility of the five-subtype classification in the same manner as reported elsewhere [8], we conducted two-step cluster analysis of the 923 patients using SPSS Statistics Base 22 software (IBM, Armonk, NY, USA). To avoid bias beset by the problem of multicollinearity, current or worst disease status was handled as a single variable (Table 2). The other variables evaluated were: sex; age of onset; disease duration; presence of thymoma; presence of thymic hyperplasia in thymectomized cases; positivity for AChR-Ab or MuSK-Ab; and positivity for other concurrent autoantibodies (Table 2).

**Table 2:** Set of variables used in the cluster analyses

Patients’ backgrounds	Autoantibody status	Disease status during the worst condition	Current disease status
Sex Age of onset Disease duration Presence of thymoma Presence of thymic hyperplasia	AChR-Ab MuSK-Ab Non-MG-specific antibodies	*One of the following:* The worst MGFA classification The worst QMG	*One of the following:* Current PIS Current QMG MG-QOL-15 score

*AChR-Ab* anti-acetylcholine receptor antibody, *MuSK-Ab* anti-muscle specific kinase antibody, *MG* myasthenia gravis, *MGFA* MG Foundation of America, *QMG* quantitative MG, *MG-QOL-15* 15-item MG-specific quality of life scale, *PIS* post-intervention status

### Early-stage response to treatment and stability of improved status in each of the five subtypes

#### Early-stage response to treatment

The time (months) from the start of the immunotherapy until achieving first MM-or-better ≥1 M was determined from medical records and compared between the five subtypes using Kaplan-Meier analysis and the log-rank test with the Cochran-Mantel-Haenszel procedure. The time required to achieve first MM-or-better ≥1 M in 50% of patients was also compared among subtypes.

#### Stability of improved status of MM-or-better ≥1 M

As an indicator of stability of improved status, the rate of the number of patients who maintained minimal manifestations in the 2015 survey/that of patients who achieved the status at least once was calculated and compared among the five subtypes.

### Statistical analysis

All statistical analyses were performed using SPSS Statistics Base 22 software (IBM) and MATLAB R2015a (MathWorks, Natick, MA, USA). All continuous data are expressed as the mean ± standard deviation (SD) and the median.

## Results

### Two-step cluster analysis

Based on the results of two-step cluster analyses, all 923 MG patients could be classified into the same five subtypes described elsewhere [8]: ocular MG; thymoma-associated MG; MG with thymic hyperplasia; AChR-Ab-negative MG; and other (in order of predicted importance). Among these five subtypes, the residual patients group “other” was the largest, and could be defined as generalized AChR-Ab-positive MG without thymic abnormalities. These results were demonstrated repeatedly with several sets of variables, as shown in Table 2, which confirmed the high reliability and reproducibility of the classification system. Although the order among thymoma-associated MG, MG with thymic hyperplasia, and AChR-Ab-negative MG was unstable depending on the variable sets used, the differences in terms of predicted importance were not large. These results were almost identical to those reported elsewhere [8], with only minor discrepancies in regard to the order of selection priority (the order in the previous study was as follows: ocular MG; MG with thymic hyperplasia; AChR-Ab-negative MG; thymoma-associated MG; and AChR-Ab-positive MG without thymic abnormalities). In the present study, the quality of clusterization under each set of variables, which was estimated using a previously reported interpretation model [14], was indicated as “fair” to “good” for all clusters, suggesting that the results were reasonable.

A total of 111 patients (10.2%) fit two of the five subtypes (Table 3). These patients were allocated to sole subtypes according to the separation priority in the two-step cluster analysis. For example, an ocular MG patient with thymoma was allocated into ocular MG. Under this criterion, the percentage of patients assigned to the five subtypes was as follows: ocular MG, 23.0%; thymoma-associated MG, 21.5%; MG with thymic hyperplasia, 12.9%; AChR-Ab-negative MG, 12.1%; and AChR-Ab-positive MG without thymic abnormalities, 30.5% (Table 4).

**Table 3:** Number of patients fitting two categories

	Number of patients	Final assignment
Ocular MG and thymoma-associated MG	32 (2.9%)	Ocular MG
Ocular MG and AChR-Ab-negative MG	56 (5.1%)	Ocular MG
Ocular MG and MG with thymic hyperplasia	8 (0.7%)	Ocular MG
MG with thymic hyperplasia and AChR-Ab-negative MG	14 (1.3%)	THMG
Thymoma-associated MG and AChR-Ab-negative MG	1 (0.09%)	TAMG

Values within the parentheses show the percentages of the total of 1,088 patients

*AChR-Ab* anti-acetylcholine receptor antibody, *MG* myasthenia gravis

**Table 4:** Characteristics and severity for each of the five MG subtypes

	Ocular MG	Thymoma- associated MG	MG with thymic hyperplasia	AChR-Ab- negative MG	(MuSK-Ab -positive)	AChR-Ab-positive MG without thymic abnormalities	Total
Patients (n)	250	234	140	132	(22)	332	1088
Female,%	52.0	67.5	81.4*	81.1*	(81.8)	61.1	65.4
Onset age, y	51.0 ± 20.0, 53.0^a^	51.0 ± 12.8, 51.0	33.3 ± 13.9, 31.0^a^	39.9 ± 16.2, 41.5^a^	(38.6 ± 15.3, 42.0)	50.7 ± 20.7, 55.0^a^	47.3 ± 18.8, 48.1
Duration of disease, y	10.4 ± 11.4, 6.4	9.5 ± 7.7, 8.0	17.4 ± 11.8, 14.5^a^	10.8 ± 9.3, 8.0	(11.0 ± 8.1, 9.8)	11.6 ± 10.5, 8.2	11.6 ± 10.6, 8.2
AChR-Ab-positivity,%	77.2	99.6	89.4	0.0	(0.0)	100.0	81.3
MuSK-Ab-positivity,%	0.0%	0.0%	0.0%	20.6%	(100.0%)	0.0%	2.1%
Thymectomy,%	23.6%*	97.4%*	100.0%*	12.1%*	(9.1%)	35.8%*	51.7%
The worst condition of the disease							
MGFA classification (*n* = 1088)							
I,%	100%	0.0%	0.0%	0.0%	(0.0%)	0.0%	23.0%
II,%	0.0%	44.0%	52.9%	59.1%	(45.5%)	64.8%	43.2%
III,%	0.0%	27.8%	30.7%	28.0%	(13.6%)	20.5%	19.6%
IV,%	0.0%	7.7%	7.9%	4.5%	(9.1%)	4.2%	4.5%
V,%	0.0%	20.5%	8.6%	8.3%	(31.8%)	10.5%	9.7%
Rate of MGFA > III,%	0.0%*	56.0%*	47.1%	40.9%	(54.5%)	35.2%	33.8%
QMG score (*n* = 922)	6.6 ± 2.6, 6.0^a^ (*n* = 225)	17.1 ± 8.0,15.5^a^ (*n* = 194)	15.8 ± 5.8, 15.0^a^ (*n* = 107)	14.7 ± 7.2, 13.0 (*n* = 114)	18.1 ± 9.7, 15.5 (*n* = 20)	14.7 ± 7.0, 13.0 (*n* = 282)	13.4 ± 7.5, 12.0
Current disease condition (mean ± SD, median)							
QMG score (*n* = 923)	4.2 ± 2.8, 4.0^a^ (*n* = 208)	6.8 ± 4.8, 6.0 (*n* = 198)	7.8 ± 5.5, 7.0 (*n* = 125)	8.4 ± 5.4, 8.0 (*n* = 106)	(8.3 ± 6.4, 7.0) (*n* = 20)	7.2 ± 4.8, 6.0 (*n* = 286)	6.6 ± 4.8, 6.0
MGC score (*n* = 923)	1.9 ± 2.5, 1.0^a^ (*n* = 208)	4.5 ± 5.4, 3.0 (*n* = 198)	5.4 ± 5.7, 3.0 (*n* = 125)	6.5 ± 6.2, 5.0^a^ (*n* = 106)	(6.2 ± 7.0, 4.5) (*n* = 20)	4.2 ± 4.6, 3.0 (*n* = 286)	4.1 ± 5.0, 3.0
MG-QOL-15 (*n* = 923)	8.1 ± 9.0, 5.0^a^ (*n* = 208)	14.7 ± 13.6, 11.0 (*n* = 198)	16.2 ± 13.7, 13.5^a^ (*n* = 125)	14.6 ± 12.6, 12.0 (*n* = 106)	(11.6 ± 8.8, 11.0) (*n* = 20)	14.1 ± 13.9, 9.0 (*n* = 286)	13.2 ± 13.0, 9.0

All continuous data are expressed as the mean ± standard deviation (SD) and the median

*AChR-Ab* anti-acetylcholine receptor antibody, *MG* myasthenia gravis, *MGC* MG composite scale, *MGFA* MG Foundation of America, *MG-QOL-15* 15-item MG-specific quality of life scale, *MuSK-Ab-positive* MG patients with serum anti-muscle specific kinase (MuSK) autoantibody in AChR-Ab-negative patients, *QMG* quantitative MG score, *SD* standard deviation

*p < 0.0001, chi-square test (compared to the others), ^†^ *p* < 0.0001, Mann–Whitney U test

MG with thymic hyperplasia is only diagnosed for thymectomized patients; therefore, some non-thymectomized patients with thymic hyperplasia may be assigned as other subtypes, particularly AChR-Ab-positive MG patients without thymic abnormalities.

### Clinical characteristics of each subtype

The clinical characteristics, including current and worst severity, for each of the five subtypes are shown in Table 4. The patients with MuSK-Ab were not separated by two-step cluster analysis because the number is not great enough for statistical evaluation. However, MG patients with MuSK-Ab showed a distinct clinical manifestation and therapy responsiveness reflecting the unique pathological mechanism [15]. Therefore, details of MG patients with MuSK-Ab (*n* = 22) are individually described next to SNMG patients in Table 4. The percentage of females was significantly higher among MG with thymic hyperplasia and AChR-Ab-negative MG patients compared with the other three subtypes (*p* < 0.0001, chi-square test). Onset age was significantly younger in MG with thymic hyperplasia and AChR-Ab-negative MG patients (*p* < 0.0001, Mann–Whitney U test) and older in ocular MG and AChR-Ab-positive MG patients without thymic abnormalities (*p* < 0.0001, Mann–Whitney U test).

Severity at the worst condition (MGFA classification and QMG) was significantly higher in thymoma-associated MG patients (*p* < 0.0001, Mann–Whitney U test). Patients with MuSK-Ab also showed worst severity at the same level as thymoma-associated MG patients, although this result was not statistically significant because of the small number of patients. The severity scales (QMG and MGC), and a QOL scale (MG-QOL-15) scores in the present survey were generally worse, although not statistically significant, in MG with thymic hyperplasia and AChR-Ab-negative MG patients, both of which primarily comprise females with younger onset ages. On the other hand, as a matter of course, ocular MG patients showed much lower clinical severity in all batteries at both current and worst conditions (*p* < 0.0001, Mann–Whitney U test).

### Onset age histograms of the five subtypes

**Fig. 1:**
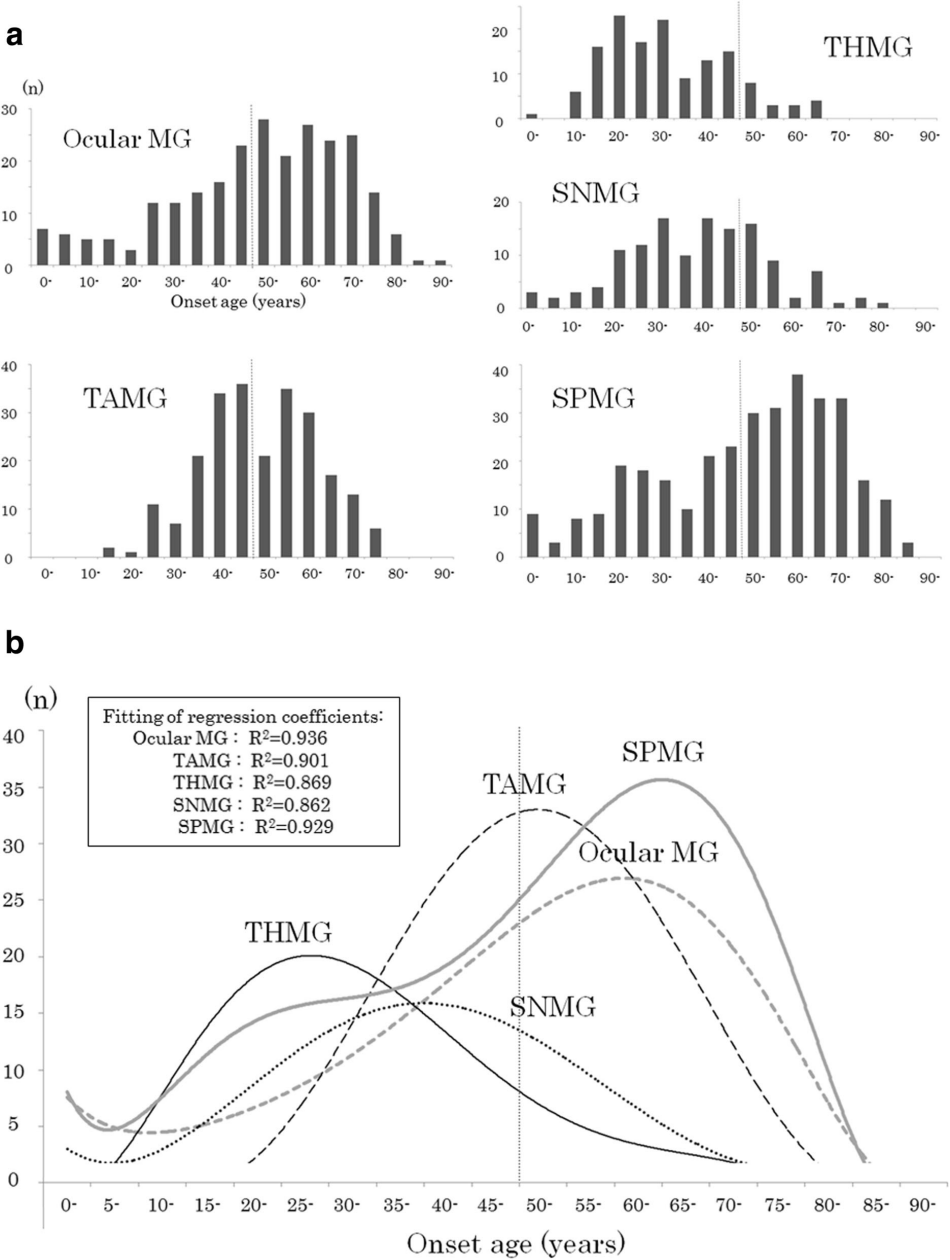
Histograms and approximate curves for onset age in the five MG subtypes. **a** Histograms for ocular MG, generalized thymoma-associated MG (TAMG), generalized MG with thymic hyperplasia (THMG), generalized AChR-Ab-negative MG (SNMG) and generalized AChR-Ab-positive MG without thymic abnormalities (SPMG). **b** Superimposed approximate curves for the five subtypes regarding the distribution of onset age. The vertical broken line indicates the cutoff onset age of 50 years between early- and late-onset MG. MG, myasthenia gravis

Histograms of the onset age for each of the five subtypes are shown in Fig. 1a. These histograms were converted into approximate curves (sixth-order polynomial approximations) and superimposed in Fig. 1b. The peak ages of the histogram were 60–64 years in ocular MG, 25–29 years in MG with thymic hyperplasia, 35–39 years in AChR-Ab-negative MG, 50–54 years in thymoma-associated MG, and 65–69 years in AChR-Ab-positive MG without thymic abnormalities. The histogram was skewed toward younger onset age in MG with thymic hyperplasia and toward older onset age in ocular MG and AChR-Ab-positive MG without thymic abnormalities. Regarding patients with MuSK-Ab (*n* = 22), the mean ± SD onset age was 38.6 ± 15.3 (median, 42.0 years), and the ratio of females was 81.8%; however, neither of these findings was significantly different from other AChR-Ab-negative MG patients.

### Early-stage response to treatment and stability of improved status among the five subtypes

Details of past immunotherapy for each of the five subtypes are shown at the top of Table 5.

**Table 5:** Details of past treatment and response to treatment for each of the five subtypes

	Ocular MG	Thymoma- associated MG	MG with thymic hyperplasia	AChR-Ab- negative MG	(MuSK-Ab -positive)	AChR-Ab-positive MG without thymic abnormalities	Total
Past immunotherapy (*n* = 923)							
Peak dose of oral PSL, mg/day	9.2 ± 12.2, 5.0^†^	28.5 ± 18.8, 30.0^†^	29.7 ± 19.4, 30.0^†^	18.8 ± 17.2, 15.0	32.6 ± 20.6, 30.0	23.7 ± 20.2, 20.0	21.5 ± 19.3, 15.0
Duration of PSL ≥20 mg/day, M	0.0 ± 0.0, 0.0^†^	12.0 ± 25.2, 5.0^†^	13.0 ± 27.3, 6.0^†^	3.8 ± 7.0, 0.0	7.2 ± 9.5, 4.0	8.2 ± 17.0, 2.0	7.9 ± 19.3, 1.0
CNIs,%	24.0%*	68.2%*	54.0%	67.4%	(72.7%)	58.1%	52.9%
PP,%	2.0%*	48.1%*	22.1%	46.0%*	(54.5%)	37.2%	27.3%
IVIG,%	6.1%*	36.1%	29.9%	42.5%*	(27.3%)	24.7%	15.0%
Initial response to treatment (*n* = 923, see Fig. 2a)							
Achievement of MM-or-better once,%	79.8%*	73.5%	66.1%	56.2%*	(75.0%)	67.8%	70.2%
Months to achieve MM-or-better in 50% of patients	4.0^‡^	8.0	18.0^‡^	31.0^‡^	(7.0)	6.0	8.0
Stability of improved status (*n* = 923)							
MM-or-better at present,%	74.0%*	58.1%	49.6%	39.6%*	(55.0%)	55.4%	57.6%
Maintaining rate of MM-or-better, %	92.7%*	79.0%	75.0%	70.5%	(73.3%)	81.7%	82.1%

All continuous data are expressed as the mean ± standard deviation (SD) and the median

*CNIs* calcineurin inhibitors, *EAT* early aggressive therapy, *IVIG* intravenous immunoglobulin, *M* months, *MG* myasthenia gravis, *MM-or-better ≥1* M minimal manifestation or better status lasting more than one month, *MuSK-Ab-positive* MG patients with serum anti-muscle specific kinase (MuSK) autoantibody in AChR-Ab-negative patients, *PP* plasmapheresis, *PSL* prednisolone, *SD* standard deviation

*p < 0.0001, chi-square test,^†^ *p* < 0.0001, Mann–Whitney U test,^‡^ *p* < 0.0001, log-rank test

#### Early-stage response to treatment (first achievement of MM-or-better ≥1 M)

As shown in the middle of Table 5, the rate of the patients achieving MM-or-better ≥1 M at least once was significantly higher for ocular MG (*p* < 0.001, chi-square test) and significantly lower for AChR-Ab-negative MG (*p* < 0.001, chi-square test).

**Fig. 2:**
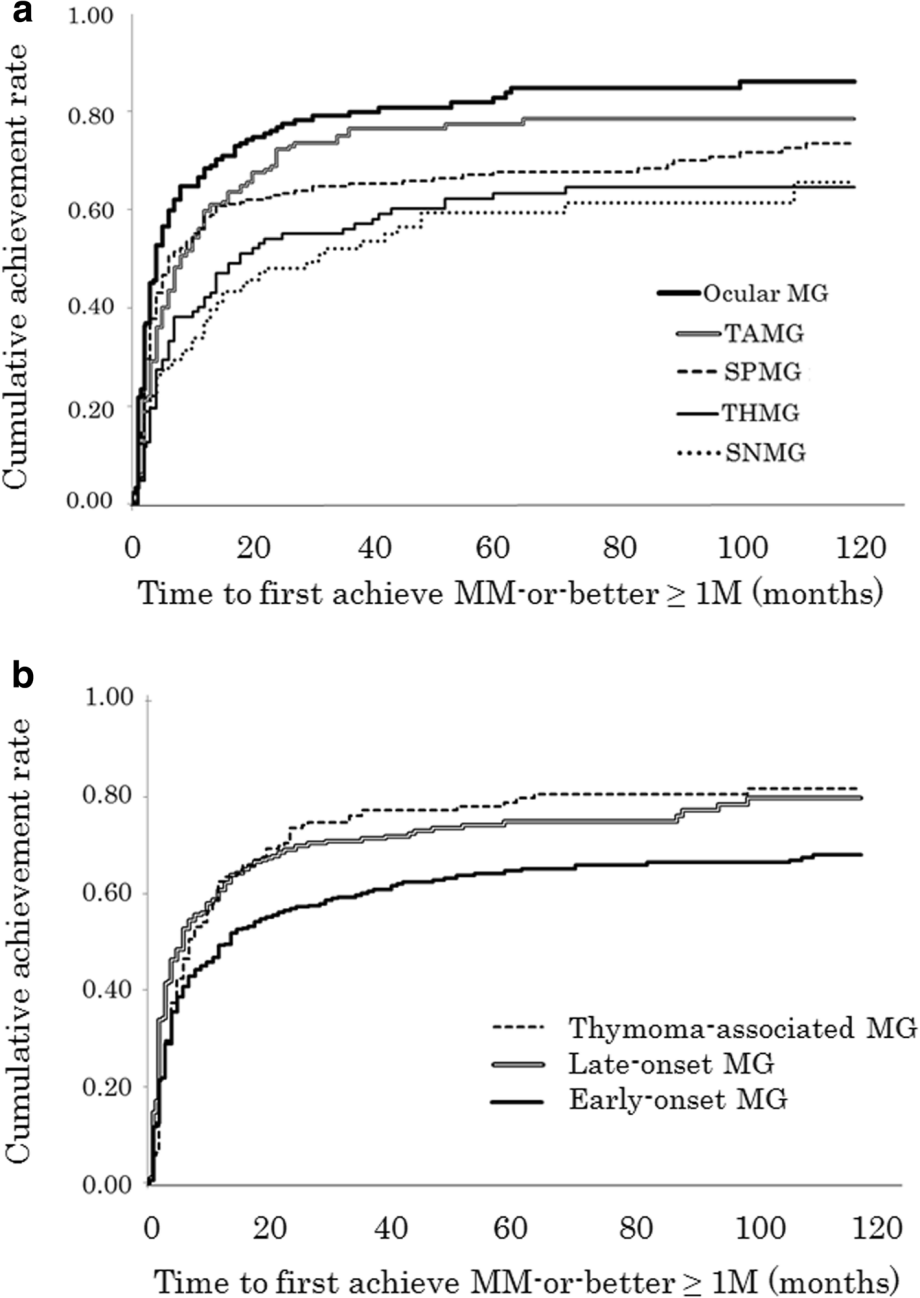
Kaplan-Meier curves for the first achievement of MM-or-better ≥1 M in the five subtypes and those in the three subtypes of early-onset, late-onset, and thymoma-associated MG. **a** Kaplan-Meier curves for the five subtypes [ocular MG, generalized thymoma-associated MG (TAMG), generalized MG with thymic hyperplasia (THMG), generalized AChR-Ab-negative MG (SNMG) and generalized AChR-Ab-positive MG without thymic abnormalities (SPMG)]. **b** Kaplan-Meier curves for the three subtypes of early-onset, late-onset, and thymoma-associated MG. MM, minimal manifestations; MG, myasthenia gravis

Kaplan-Meier curves for the time to first achieve MM-or-better ≥1 M in each of the five subtypes up to 10 years from initiating immunotherapy are shown in Fig. 2a. The time to first achieve MM-or-better ≥1 M was significantly different among the five subtypes (*p* < 0.0001; generalized Wilcoxon test and log-rank test). Significant differences were observed between all pairs of two subtypes (*p* < 0.01 for all pairs; generalized Wilcoxon test) except MG with thymic hyperplasia and AChR-Ab-negative MG (*p* ≥ 0.10). Patients with ocular MG showed the best early-stage response to treatment compared with others (*p* < 0.0001; log-rank test, *p* < 0.001; chi-square test). The time required to achieve MM-or-better ≥1 M in 50% of the patients was significantly longer in MG with thymic hyperplasia and AChR-Ab-negative MG compared with ocular MG, thymoma-associated MG, and AChR-Ab-positive MG without thymic abnormalities (*p* < 0.0001, log-rank test; middle of Table 5).

For comparison, Kaplan-Meier curves for the time to first achieve MM-or-better ≥1 M in early-onset, late-onset, and thymoma-associated MG (three-type classification) are shown in Fig. 2b. Significant differences were observed between early- and late-onset MG (*p* < 0.01) and between early-onset and thymoma-associated MG (*p* < 0.01); however, no significant differences were found between late-onset and thymoma-associated MG (*p* ≥ 0.10).

#### Stability of improved status

The rates of patients with MM-or-better status during the survey and stability of improved status are shown in the bottom of Table 5. Stability of improved status was significantly better in ocular MG compared with other subtypes (*p* < 0.0001; chi-square test); however, no significant differences were observed among subtypes other than ocular MG (*p* ≥ 0.10 for all pairs, excluding ocular MG; chi-square test).

## Discussion

The present analyses based on several sets of variables classified 923 MG patients into the same five following subtypes with the same characteristics of the onset-age histograms as reported in our previous study [8]: ocular MG (AChR-Ab-positivity, 77%; histogram of onset age, skewed to older age); thymoma-associated MG (100%; normal distribution); MG with thymic hyperplasia (89%; skewed to younger age); AChR-Ab-negative MG (0%; normal distribution); and AChR-Ab-positive MG without thymic abnormalities (100%, skewed to older age). The results from the two different samples demonstrated high reproducibility, which suggests the reliability of our five-subtype classification method. In the process of analyses, two points were suggested. First, discrimination between ocular and generalized MG is more principal than that according to onset age, thymus pathology or AChR-Ab-positivity. Second, AChR-Ab-negative MG shows normal distribution of onset age not fitting discrimination based on onset age. Therefore, it is probably better to adopt the often-used three-type classification (early-onset, late-onset and thymoma-associated MG) for generalized and AChR-Ab-positive phenotypes. Consistently, in our five-subtype classification, MG with thymic hyperplasia (with early-onset age), AChR-Ab-positive MG without thymic abnormalities (with late-onset age) and thymoma-associated MG were generalized and AChR-Ab-positive phenotypes.

In fact, these results of our classification statistically performed are consistent with a recently reported classification of MG by Gilhus et al. [16, 17], which included the following classifications: early-onset MG; late-onset MG; thymoma-associated MG; MuSK-Ab positive MG; lipoprotein-related protein 4 (LRP4)-Ab positive MG; seronegative MG; and ocular MG. In addition, they commented that early- and late-onset MG should be distinguished according to onset age only for patients having generalized symptoms and AChR-Ab. In the present study, because of their small numbers, MuSK-Ab-positive MG patients were not separated, and LRP4-Ab positivity was not systematically determined; though MG patients with MuSK-Ab or LRP 4-Ab have a distinct clinical manifestation and a unique pathological mechanism [15].

Ocular MG was found to have unique characteristics such as having a higher onset age, predominantly affecting males, and having an ocular muscle-specific pathogenesis [18], which may be related to the aging-associated susceptibility of ocular muscles to antibodies against the neuromuscular junction. Given that response to treatment and stability of improved status were substantially better in ocular MG compared with the other four subtypes, it seems reasonable to conclude that ocular MG should be treated as a distinct subgroup of MG in the clinical setting.

Among the four generalized subtypes in the present classification method, both early-stage response to treatment and stability of improved status were worst in AChR-Ab-negative MG, although symptoms at the time the condition was at its worst were not particularly severe. Patients with MuSK-Ab-positive MG showed better results despite having more severe worst conditions, which suggests that AChR-Ab-negative MG, excluding MuSK-Ab-positive MG, is distinct from other generalized MG subtypes from the perspective of response to therapy. Overall, as shown in Fig. 2a, each of the five present subtypes showed different levels of response to treatment, whereas such differences in the three commonly used subtypes (early-onset, late-onset, and thymoma-associated MG) remain somewhat unclear (Fig. 2b). It would be more helpful in the clinical setting to elucidate the levels of response to some types of medication or therapy (e.g. corticosteroids, non-steroid immunosuppressants, intravenous immunoglobulin and plasmapheresis) in the five subtypes. However, it was difficult to analyze such response levels, as plural treatment agents and methods were employed simultaneously in most of individual patients. We are now analyzing the response levels according to patterns of immune treatment (treatment strategies) in generalized MG patients [19]. Such analysis should be performed also for the present five subtypes, but could not be addressed in the present report.

The present study did have some limitations. First, 331 (30.4%) of the 1,088 patients were included in our previous survey in 2012, which might have affected the reproducibility of the present five-subtype classification. Second, almost all of the MG patients in our database are Japanese; therefore, a race/ethnicity bias may have affected the results. Finally, some MG patients with thymic hyperplasia might have been classified as AChR-Ab-positive MG without thymic abnormalities because the diagnosis of thymic hyperplasia is made based on the results of pathological examinations after thymectomy. However, the frequency of thymectomy for MG patients without thymoma has been decreasing [20]; therefore, some of the AChR-Ab-positive MG patients with younger onset age who had not undergone thymectomy could have had thymic hyperplasia.

## Conclusion

The results of the present study suggest that MG patients can be classified into the following five subtypes in order of priority: ocular MG; thymoma-associated MG; MG with thymic hyperplasia; AChR-Ab-negative MG; and AChR-Ab-positive MG without thymic abnormalities. All MG patients can be allocated to one of the subtypes based on the results of routine examinations. These five subtypes were shown to have characteristic demographic characteristics, clinical severity, and therapeutic responses. Therefore, our five-subtype classification method is expected to be beneficial not only for elucidating disease types, but also for planning proper treatment for individual patients.

## Abbreviations

AChR-Ab: Anti-acetylcholine receptor antibody
LRP4: Lipoprotein-related protein 4
MG: Myasthenia gravis
MGC: MG Composite
MGFA: MG Foundation of America
MM: Minimal manifestations
MuSK: Muscle specific tyrosine kinase
PSL: Prednisolone
QMG: Quantitative MG score
